# ﻿Three new *Pyrenula* species with 3-septate ascospores with red or orange oil when over-mature (Ascomycota, Pyrenulales, Pyrenulaceae) from China

**DOI:** 10.3897/mycokeys.102.113619

**Published:** 2024-02-12

**Authors:** Mingzhu Dou, Shengnan Liu, Jiechen Li, André Aptroot, Zefeng Jia

**Affiliations:** 1 College of Life Sciences, Liaocheng University, Liaocheng 252059, China Liaocheng University Liaocheng China; 2 Laboratório de Botânica, Liquenologia, Instituto de Biociências, Universidade Federal de Mato Grosso do Sul, Avenida Costa e Silva s/n, Bairro Universitário, CEP 79070-900, Campo Grande, Mato Grosso do Sul, Brazil Universidade Federal de Mato Grosso do Sul Mato Grosso do Sul Brazil

**Keywords:** morphology, new species, phylogeny, Pyrenulaceae, taxonomy

## Abstract

The lichenised fungal genus *Pyrenula* is a very common crustose lichen element in tropical to subtropical forests, but little research has been done on this genus in China. During our study on *Pyrenula* in China, based on morphological characteristics, chemical traits and molecular phylogenetic analysis (ITS and nuLSU), three new 3-septate species with red or orange oil in over-mature ascospores were found: *Pyrenulainspersa***sp. nov.**, *P.thailandicoides***sp. nov.** and *P.apiculata***sp. nov.** Compared to the known 3-septate species of *Pyrenula* with red or orange oil, *P.inspersa* is characterised by the inspersed hamathecium; *P.thailandicoides* is characterised by the IKI+ red hamathecium and the existence of an unknown lichen substance; and *P.apiculata* is characterised by the absence of endospore layers in the spore tips and the absence of pseudocyphellae. It is reported for the first time that the presence of a gelatinous halo around the ascospores of *Pyrenula* is common. A word key for the *Pyrenula* species with red or orange oil in over-mature ascospores is provided.

## ﻿Introduction

The lichen genus *Pyrenula* Ach. (Pyrenulaceae) was first established by Acharius, with *Pyrenulanitida* (Weigel) Ach. as the type species (Acharius 1814). *Pyrenula* is mainly a tropical and subtropical genus (Mendonça et al. 2016) and the Neotropics are the centre of diversity of the genus, which typically grow on bark (Aptroot 2012). The genus is characterised by UV– or UV+ yellow thallus, with or without pseudocyphellae, with or without lichexanthone or anthraquinones, perithecioid ascomata, occasionally inspersed hamathecia, unbranched filaments and distoseptate, transversely septate or (sub)muriform ascospores (Aptroot 2012; Mendonça et al. 2016).

In a world key of *Pyrenula* species, Aptroot (2012) accepted 169 species out of the ca. 745 named taxa in the genus. Since then, many new species of *Pyrenula* have been described and the genus now comprises ca. 238 species (Aptroot 2012, 2021; Aptroot et al. 2018; Ingle et al. 2018; Miranda-González et al. 2022; Mishra et al. 2022; Lücking et al. 2023; Soto-Medina et al. 2023), of which 41 species have so far been found in China (Aptroot 2012, 2021; Wang et al. 2018; Fu et al. 2018, 2019; Wei 2020; Xie et al. 2021).

Harris (1989) was the first to recognise the presence of red or orange oily granules in over-mature ascospores of some *Pyrenula* species and to point out the significance of the degradation stage of spores for the taxonomy of *Pyrenula*. Aptroot et al (2013) described the degradation process in detail: in a few species, the old spores assume a reddish tinge, the wall becomes red-brown and the remains of the lumina develop into red or orange granules. Now, a total of seven species with red or orange oil in over-mature ascospores have been reported, of which four have transverse distoseptate ascospores, viz. *P.concastroma* R.C. Harris, *P.bahiana* Malme, *P.sexlocularis* (Nyl.) Müll. Arg. and *P.thailandica* Aptroot; three have (sub)muriform ascospores, viz. *P.seminuda* (Müll. Arg.) Sipman & Aptroot, *P.breutelii* (Müll. Arg.) Aptroot and *P.macularis* (Zahlbr.) R.C. Harris. Our study adds three septate *Pyrenula* species with red or orange oily granules in over-mature ascospores.

As far as we can tell, there have been no reports of a gelatinous halo around the ascospores in *Pyrenula*. This could mislead lichen taxonomists into believing that ascospore gelatinous haloes are absent in this genus. However, during our study of *Pyrenula* in southern China, we found that gelatinous haloes are common in this genus and present in all the three new species described here.

In term of molecular data, the attempts to infer relationships within Pyrenulaceae presented two well-supported groups that do not seem to differ based on their morphology, apart from the presence/absence of pseudocyphellae; meanwhile, delimitation problems in few taxa, for instance, *P.quassiicola* and *P.mamillana*, were highlighted (Weerakoon et al. 2012; Gueidan et al. 2016). Our phylogenetic analysis using ribosomal genes (nuLSU and ITS) confirmed the above conclusions and supported the description of three new species.

## ﻿Materials and methods

### ﻿Morphological and chemical analyses

The specimens were collected in southern China and deposited in the Fungarium, College of Life Sciences, Liaocheng University, China (**LCUF**). Morphological and anatomical characters of thalli and apothecia were examined and photographed under an Olympus SZX16 dissecting microscope and an Olympus BX53 compound microscope. The lichen secondary metabolites were detected and identified by thin-layer chromatography using solvent C and B (Orange et al. 2010; Jia and Wei 2016).

### ﻿DNA extraction, PCR sequencing and phylogenetic analysis

Genomic DNA was extracted from ascomata using the Hi-DNA-secure Plant Kit (Tiangen, Beijing, China) according to the manufacturer’s protocol. The nuLSU and ITS regions were amplified using the primer pair AL2R/LR6 (Vilgalys and Hester 1990; Mangold et al. 2008) and ITS1F/ITS4 (White et al. 1990; Gardes and Bruns 1993). The PCR amplification progress followed Dou et al. (2018) and the PCR products were sequenced by Biosune Inc. (Shanghai). The newly-generated sequences were submitted to GenBank (Table 1).

**Table 1.** Information for the sequences used in this study. Newly-generated sequences are shown in bold.

Species Name	Specimen No.	Locality	GenBank accession number	
			ITS	nuLSU
*P.thailandicoides* M.Z. Dou & Z.F. Jia	FJ220208	China Fujian	**OR578593**	—
	YN18212	China Yunnan	**OR578589**	**OR578570**
	YN18015	China Yunnan	**OR578590**	**OR578571**
*P.inspersa* M.Z. Dou & Z.F. Jia	HN17058	China Hainan	**OR578591**	**OR578572**
*P.apiculata* M.Z. Dou & Z.F. Jia	YN18172	China Yunnan	**OR578592**	**OR578573**
P.cf.acutalis R.C. Harris	F_19092_b	Australia	—	DQ329026
P.aff.aggregataspistea Aptroot & M. Cáceres	AA11618	Brazil	—	KT808561
*P.aggregataspistea* Aptroot & M. Cáceres	AA11216	Brazil	KT820112	KT808557
*P.anomala* (Ach.) A. Massal.	AA11222	Brazil	KT820168	KT808607
	AA11607	Brazil	KT820116	—
	AA15591	Brazil	KT820113	—
*P.arthoniotheca* Upreti	AA11887	Brazil	KT820120	—
*P.aspistea* (Ach.) Ach	AA11263	Brazil	KT820121	KT808560
	AA13547	Brazil	KT820123	—
	CBS_109078	Hong Kong	—	EF411063
	CG3030	Vietnam	KT820124	KT808562
	CG3060	Vietnam	KT820125	KT808564
	CG3070	Vietnam	KT820126	—
	CG3071	Vietnam	KT820127	—
	GW1042	Sri Lanka	JQ927450	JQ927469
	GW1044	Sri Lanka	JQ927451	JQ927470
	RAMK17271	Thailand	KT820128	—
	RAMK17277	Thailand	KT820129	KT808563
*P.astroidea* (Fée) R.C. Harris	RAMK17281	Thailand	KT820088	—
*P.bahiana* Malme	RVH1	Laos	KT820090	—
	RVH2	Laos	KT820091	KT808614
	RVH3	Laos	KT820092	KT808605
*P.balia* (Kremp.) R.C. Harris	CG3063	Vietnam	KT820130	KT808566
*P.brunnea* Fée	CG3023	Vietnam	KT820093	—
P.cf.subglabrata (Nyl.) Müll. Arg	CG3028	Vietnam	KT820140	KT808574
*P.chlorospila* (Nyl.) Arnol	CG1520b	England	JQ927452	JQ927471
*P.cornutispora* Aptroot & M. Cáceres	AA11938	Brazil	KT820131	KT808618
	ISE_AA11938	Brazil	NR_158911	NG_060160
*P.corticata* (Müll. Arg.) R.C. Harris	AA11443	Brazil	KT820132	KT808568
	AA11466	Brazil	KT820133	KT808569
*P.confinis* (Nyl.) R.C. Harris	AA13575	Brazil	—	KT808567
*P.cruenta* (Mont.) Vain	Green_PYCR12	USA	KC592268	—
	Green_PYCR16	USA	KC592269	—
	Green_PYCR4	USA	KC592267	—
	Lutzoni_9806174	Puerto Rico	—	AF279407
*P.fetivica* (Kremp.) Müll. Arg	CG1963	Vietnam	KT820134	—
*P.fetivica* (Kremp.) Müll. Arg	GW307A	Sri Lanka	JQ927453	JQ927472
	GW835	Sri Lanka	JQ927454	—
*P.infraleucotrypa* Aptroot & M. Cáceres	AA11105	Brazil	KT820114	KT808558
	AA11468	Brazi	KT820136	—
	AA11499	Brazi	KT820115	—
	AA15450	Brazi	KT820142	KT808575
	AA15451	Brazi	KT820117	KT808559
*P.inframamillana* Aptroot & M. Cáceres	AA11220	Brazi	KT820137	KT808572
	AA11272	Brazi	KT820138	KT808571
	AA11897	Brazi	KT820139	KT808573
*P.laevigata* (Pers.) Arnold	OL_206758	Norway	MK812685	—
	OL_206773	Norway	MK812185	—
	Palice 5608	Slovakia	—	AY607736
P.cf.leucostoma Ach.	F_19082	Australia	—	DQ329024
*P.macrospora* (Degel.) Coppins & P. James	CG1520a	England	JQ927455	JQ927473
*P.mamillana* (Ach.) Trevis.	AA11342	Brazil	KT820143	KT808576
	AA11610	Brazil	KT820144	KT808615
	AA11846	Brazil	KT820145	KT808617
	AA15465	Brazil	KT820146	KT808579
	CG3014	Vietnam	KT820147	KT808580
	CG3034	Vietnam	KT820149	KT808582
	CG3058	Vietnam	KT820150	KT808583
	CG3059	Vietnam	KT820151	KT808584
P.aff.mamillana (Ach.) Trevis.	GW818A	Sri Lank	JQ927456	JQ927474
*P.massariospora* (Starbäck) R.C. Harris	CG3061	Vietnam	KT820153	KT808585
	CG3062	Vietnam	KT820154	KT808586
	GW1028	Sri Lanka	JQ927457	JQ927475
*P.minor* Fée	AA11505	Brazil	KT820155	KT808620
	AA13516	Brazil	—	KT808587
*P.minutispora* Aptroot & M. Cáceres	AA11877	Brazil	KT820119	—
	ABL_AA11877	Brazil	NR_136140	—
*P.nitida* (Weigel) Ach.	17076	Poland	MN387114	—
	17081	Poland	MN387115	—
	17146	Poland	MN387116	—
	17189	Poland	MN387117	—
	F_5929	Czech Republic	JQ927458	DQ329023
	s. n.	Germany	—	AY607737
*P.nitidella* (Flörke) Müll. Arg.	17082	Poland	MN387139	—
	CG3027	Vietnam	KT820156	—
*P.occidentalis* (R.C. Harris) R.C. Harris	OL_206777	Norway	MK811633	—
*P.ochraceoflava* (Nyl.) R.C. Harris	Gaya_160308_EGB11	USA	KC592275	—
*P.punctella* (Nyl.) Trevis.	Tripp4522	—	KT232213	—
*P.pyrenuloides* (Mont.) R.C. Harris	CG1545	Vietnam	KT820094	—
*P.quassiicola* Fée	CG3001	Vietnam	KT820098	KT808588
	CG3019	Vietnam	KT820101	KT808591
	CG3032	Vietnam	KT820104	KT808592
	CG3033	Vietnam	KT820105	KT808593
	RVH6	Laos	KT820107	KT808595
*P.sanguinea* Aptroot, M. Cáceres & Lücking	15707F	Brazil	—	KF697129
*P.leucostoma* Aptroot & Gueidan	AFTOL_ID387	USA	DQ782845	—
	DUKE_0047599	—	NR_119610	NG_068722
	Reeb VR 14 VI 025	USA	—	AY640962
*P.reginae* E.L. Lima, Aptroot & M. Cáceres	ELL0010	Brazil	—	KT808596
*P.rubronitidula* Aptroot & M. Cáceres	AA11332	Brazil	KT820157	KT808597
	AA15603	Brazil	KT820158	—
	AA11697	Brazil	KT820159	KT808616
	ISE_AA11697	Brazil	NR_158913	NG_06015
*P.scutata* (Stirt.) Zahlbr	CG1635	Vietnam	KT820160	KT808598
*P.septicollaris* (Eschw.) R.C. Harris	AA13534	Brazil	KT820166	KT808610
	AA13546	Brazil	KT820161	—
	AA13555	Brazil	KT820167	—
	AA15009	Brazil	—	KT808599
	AA15012	Brazil	KT820162	KT808600
	AA15021	Brazil	KT820163	KT808601
	AA15023	Brazil	KT820164	KT808602
	AA15038	Brazil	—	KT808603
	AA15042	Brazil	KT820165	KT808604
*P.sexlocularis* (Eschw.) R.C. Harris	RAMK17261	Thailand	KT820108	KT808606
*P.* sp.	F19113n	Australia	—	DQ329027
	CG3009	Vietnam	KT820110	KT808611
	F19082r	Australia	JQ927461	DQ329025
	LHD210	Vietnam	AB935436	—
*P.subelliptica* (Tuck.) R.C. Harris	RVH5	Laos	KT820106	KT808594
*P.subglabrata* (Nyl.) Müll. Arg.	CG3069	Vietnam	KT820169	KT808608
*P.subpraelucida* Müll. Arg.	F_17550_f	Costa Rica	—	DQ329015
*P.thelemorpha* Tuck.	F_19082	Australia	JQ927460	—
*P.viridipyrgilla* Aptroot & M. Cáceres	AA11864	Brazil	KT820170	KT808619
	ISE_AA11864	Brazil	NR_158914	—
*Cyphellophoraeuropaea* (de Hoog, Mayser & Haase) Réblová & Unter.	CBS129_96	—	EF551553	FJ358248
*Endocarponpusillum* Hedw.	CG470	—	JQ927447	EF643754

Multi-locus (ITS and nuLSU) phylogenetic analysis was performed. The combined analysis included 187 sequences (Table 1), of which nine sequences were newly generated and 178 were downloaded in GenBank (Lutzoni et al. 2001; Geiser et al. 2006; Weerakoon et al. 2012; Gueidan et al. 2016). The dataset represented 121 taxa, amongst which two out-group species, *Endocarponpusillum* and *Cyphellophoraeuropaea*, were chosen, based on previous studies (Weerakoon et al. 2012; Gueidan et al. 2016). All *Pyrenula* taxa that could be found in GenBank were included in our data matrix.

The alignment of sequences for each marker (ITS and nuLSU) was undertaken independently by applying MAFFT 7 (Katoh and Standley 2013). We used the “maskSegment” function in the R package AlignmentFilter (Zhang et al. 2023) to mask ambiguously-aligned or overly-divergent segments (stringency-controlling parameter prob set to 0.05) and then used the “degap” function to remove sites with more than 50% gaps. The congruence of the two datasets was tested using a 70% reciprocal bootstrap criterion (Mason-Gamer and Kellogg 1996): the two matrices (nuLSU, ITS) were analysed separately with RAxML v.8.2.12 (Stamatakis 2014) using 100 bootstrap pseudoreplicates and implementing a GTRGAMMA model on the CIPRES Web Portal (http://www.phylo.org). The resulting trees were compared and any hard conflicts detected were eliminated by pruning sequences or taxa out of the datasets. The two single-locus alignments were concatenated in PhyloSuite v.1.2.2 (Zhang et al. 2020). The concatenated data matrix comprised 1581 characters (674 for ITS and 907 for nuLSU). For BI (Bayesian Inference) analysis, PartitionFinder 2 (Lanfear et al. 2017) was used to determine the best-fit model for each partition. The dataset was partitioned into gene groups, with the GTR+I+G and SYM+I+G substitution models applied to ITS gene and nuLSU gene, respectively. BI analysis was performed with MrBayes 3.2.7 (Ronquist et al 2012). Two runs of four chains were carried out for 10,000,000 generations and trees were sampled every 1000 generations. The convergence of parameters was checked with the programme Tracer v.1.6 (Rambaut et al. 2014). The first 25% of the convergence runs were discarded as burn-in. Construction of the ML (Maximum Likelihood) tree was undertaken by applying RAxML v.8.2.12 (Stamatakis 2014), using 100 bootstrap pseudoreplicates and a GTRGAMMA model on the CIPRES Web Portal (http://www.phylo.org). ML bootstrap values (BS) ≥ 70% and Bayesian posterior probabilities (PP) ≥ 0.95 were considered as significantly supported. The datasets/alignments were deposited in TreeBase (http://purl.org/phylo/treebase/phylows/study/TB2:S31046).

## ﻿Results and discussion

### ﻿Phylogenetic analyses

**Figure 1.**
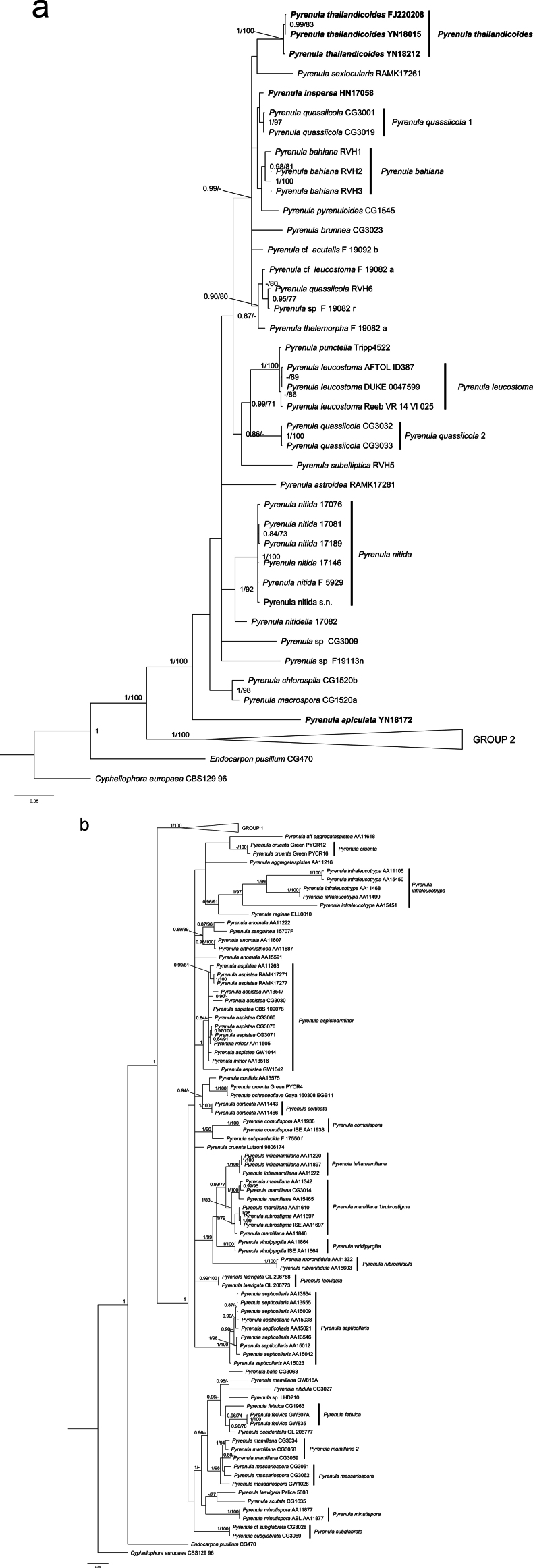
Phylogeny of the family Pyrenulaceae, based on a two-gene dataset (ITS and nuLSU) and 121 taxa **a** overview of the entire tree and details of Group 1 **b** details of Group 2. Most likely tree obtained using MrBayes. Support values are reported above the branches [posterior probability (PP)/bootstrap value (BS)]. Only significant values (higher than 95% PP and higher than 70% BS) are shown. *Cyphellophoraeuropaea* and *Endocarponpusillum* are the out-group taxa.

The dataset includes 105 ITS sequences and 82 LSU sequences, of which five ITS sequences and four LSU sequences are newly generated in this study. The BI and ML trees showed similar topologies, so only the BI tree is provided here as Fig. 1. Compared with the dataset of Gueidan et al. (2016), our phylogenetic analysis includes nine additional species (*Pyrenulapunctella*, *P.nitidella*, P.cf.acutalis, P.cf.leucostoma, *P.sanguinea*, *P.occidentalis* and three new species) and confirms the presence of two main well-supported monophyletic groups in accord with the presence/absence of pseudocyphellae as shown in Weerakoon et al. (2012) and Gueidan et al. (2016). Our phylogenetic results also indicate that delimitation problems affect several taxa, for example, *P.mamillana*, *P.quassiicola* and *P.rubrostigma*, which is consistent with Gueidan et al. (2016).

The three specimens of *Pyrenulathailandicoides* form a well-supported monophyletic group (1/100 and 0.99/83). *Pyrenulathailandicoides* is sister to *P.sexlocularis*, but with very low support (0.52/-, Suppl. material 1). *Pyrenulainspersa* is sister to *P.quassiicola* clade 1 with low support (0.79/-) and *P.apiculata* forms the first diverging lineage in Group 1 with strong support (1/100). The three new species all belong to Group 1.

### ﻿Taxonomy

#### ﻿1. Pyrenula inspersa

Taxon classificationFungiPyrenulalesPyrenulaceae

M.Z. Dou & Z.F. Jia sp. nov.

0C60A5DA-2CD2-5330-A20C-AF9E3D024FA6

Fungal Names: FN 571675

Fig. 2

##### Diagnosis.

The new species can be distinguished from the most similar species *Pyrenulathailandica* Aptroot by the hamathecium densely inspersed with minute granules and colourless oil droplets.

##### Type.

China. Hainan Province: Changjiang County, Bawangling Nature Reserve, Yajia, 19°05′07′′N, 109°07′25′′E, alt. 444 m, on bark, 10 December 2017, X.H. Wu HN17058 (LCUF:holotype: HN17058; GenBank OR578591 for ITS and OR578572 for LSU).

##### Description.

***Thallus*** corticolous, crustose, brown, surface dull, uneven, corticate with pseudocyphellae, UV-. Ascomata perithecioid, emergent, dispersed, aggregated occasionally when crowded, hemispherical, 1–1.5 mm diam., with crystals, KOH-. ***Ostioles*** apical. ***Hamathecium*** heavily inspersed with minute granules and colourless oil droplets (close-up in Suppl. material 2), IKI-. ***Ascospores*** 8 per ascus, irregularly biseriate, with gelatinous halo before becoming old, 3-septate, 28.5–50 × 10–20 μm; middle lumina diamond-shaped, end lumina triangular, with a thick layer of endospore in the spore tips; hyaline when young, brown when mature, over-mature ascospores with orange oil.

**Figure 2.**
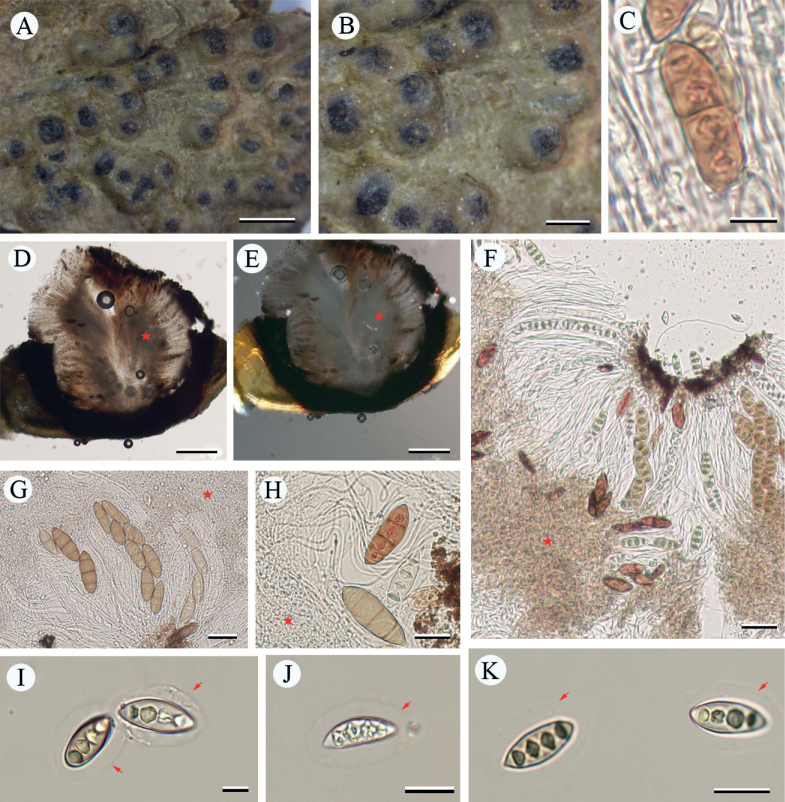
*Pyrenulainspersa* (LCUF HN17058) **A** thallus with apothecia **B** apothecia and pseudocyphellae **C, F–H** ascospores at different developmental stages, over-mature ascospores with orange-oil can be seen in **C, F** and **H D** section of apothecium **E** section visualised with polarised light showing cortex of apothecium with crystals, red stars in **D–H** show the inspersion in hamathecium **I–K** young ascospores, red arrows show gelatinous halo. Scale bars: 2 mm (**A**); 1 mm (**B**); 10 μm (**C, I**); 200 μm (**D, E**); 50 μm (**F, H**); 35 μm (**G**); 20 μm (**J, K**).

##### Chemistry.

Thallus K-, C-, KC-, UV-, hamathecium IKI-.

##### Ecology and distribution.

The new species is currently only known from the tropical regions of southern China on bark.

##### Etymology.

The specific epithet *inspersa* refers to the inspersed hamathecium.

##### Note.

This new species is similar to *Pyrenulathailandica*, *P.bahiana* and *P.concastroma* in having 3-septate ascospores with red or orange oil when over-mature. It differs from *P.thailandica* by an inspersed hamathecium and larger ascomata, which are in the latter species 0.6–1.1 mm wide (Aptroot 2012; Aptroot et al. 2012, 2013; Ingle et al. 2018). This new species differs from *P.bahiana* by larger ascospores, which are in the latter species 26–33(–35) × 10–13(–15) μm (Malme 1929; Aptroot 2012; Aptroot et al. 2013; Ingle et al. 2018). *Pyrenulaconcastroma* differs from the new species by the mostly aggregated ascomata with fused walls, but separate ostioles (Aptroot 2012; Schumm and Aptroot 2021). Although *P.quassiicola* and *P.pyrenuloides* are phylogenetically close to this new species, they can be distinguished easily by the morphology. *P.quassiicola* has smaller ascomata (0.3–0.7 mm), smaller ascospores (28–35 (–40) × 12–16 μm) containing colourless oil when over-mature and not inspersed, IKI+ (orange) hamathecium (Harris 1989). *P.pyrenuloides* has smaller ascomata (0.5–1.0 mm), larger ascospores (50–62 × 18–24 μm) containing no oil when over-mature and not inspersed, IKI+ (orange) hamathecium (Harris 1989).

#### ﻿2. Pyrenula thailandicoides

Taxon classificationFungiPyrenulalesPyrenulaceae

M.Z. Dou & Z.F. Jia sp. nov.

B156A89A-4B92-5989-9958-825D0259E12C

Fungal Names: FN 571676

Fig. 3

##### Diagnosis.

The new species can be distinguished from the most closely-related species *Pyrenulathailandica* by the IKI+ red hamathecium and an unidentified lichen substance.

##### Type.

China. Yunnan Province: Mengla County, Xishuangbanna Tropic Botanical Garden, Chinese Academy of Sciences, Rainforest Valley, 21°54′51′′N, 101°11′28′′E, alt. 626 m, on bark, 26 January 2018, X.H. Wu YN18212 (LCUF: holotype: YN18212; GenBank OR578589 for ITS and OR578570 for LSU).

##### Description.

***Thallus*** corticolous, crustose, olive-green, corticate with few pseudocyphellae, UV-. Ascomata perithecioid, emergent, dispersed, conical, 0.8–1.6 mm diam., with crystals, KOH-. ***Ostioles*** apical, white, 0.25–0.45 mm. ***Hamathecium*** not inspersed (close-up in Suppl. material 3), IKI+/I+ red (Fig. 2 and Suppl. material 4). ***Ascospores*** 8 per ascus, irregularly biseriate, with gelatinous halo before becoming old, 3-septate, (30–)35–55 × (12–)15–23 μm; middle lumina diamond-shaped, end lumina triangular, with a thick layer of endospores in the spore tips; hyaline when young, reddish-brown when mature, over-mature ascospores with red oil.

**Figure 3.**
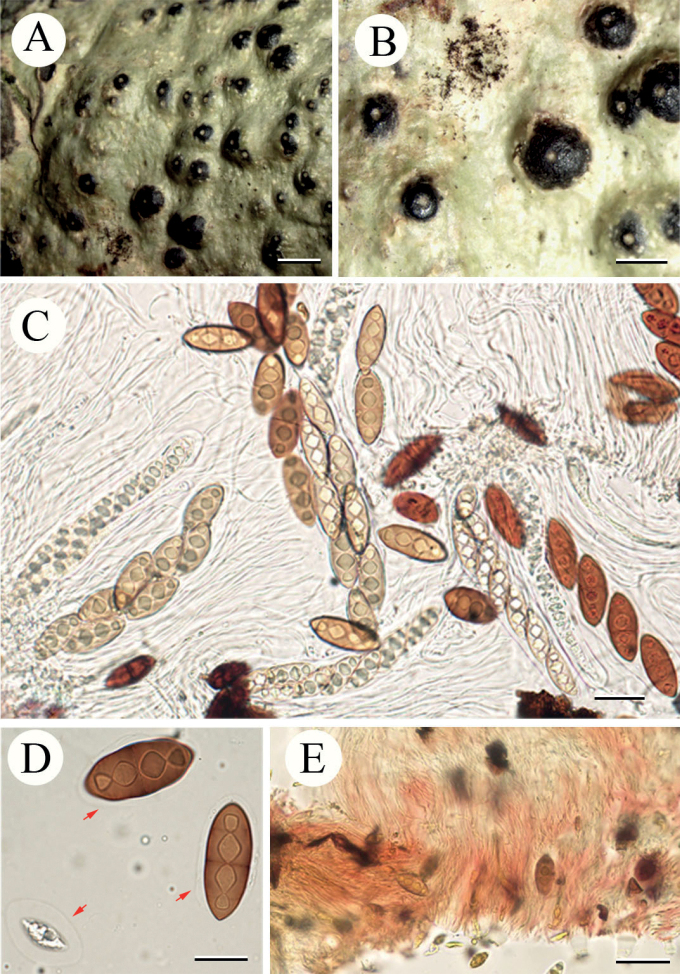
*Pyrenulathailandicoides* (LCUF YN18212) **A, B** thallus with apothecia **C, D** ascospores at different developmental stages, over-mature ascospores with red-oil can be seen in **C**, red arrows in **D** show gelatinous halo **E** IKI+ red hamathecium. Scale bars: 2 mm (**A**); 1 mm (**B**); 30 μm (**C**); 20 μm (**D**); 50 μm (**E**).

##### Chemistry.

Thallus K+ orange–brown, C-, KC+ yellow, UV-, hamathecium IKI+ red, TLC showed an unidentified substance at Rf four of solvent C (Suppl. material 5).

##### Ecology and distribution.

The new species is currently only known from the tropical and subtropical regions of southern China on bark.

##### Etymology.

The specific epithet *thailandicoides* refers to the similarity to *Pyrenulathailandica*.

##### Additional specimens examined.

China. Yunnan Province: Mengla County, Xishuangbanna Tropic Botanical Garden, Chinese Academy of Sciences, 21°55′37′′N, 101°15′27′′E, alt. 555 m, on bark, 25 January 2018, X. Zhao YN18015 (LCUF; YN18015; GenBank OR578590 for ITS and OR578571 for LSU). China. Fujian Province: Longyan City, Dongxiao National Forest Park, Frog Stone, 24°58′07′′N, 117°01′14′′E, alt. 679 m, on bark, 12 July 2022, Z.G. Ma FJ220208 (LCUF; GenBank OR578593 for ITS).

##### Notes.

This new species is similar to *Pyrenulathailandica*, *P.bahiana* and *P.concastroma* in having 3-septate ascospores with red or orange oil when over-mature. The colour reaction of hamathecium of *Pyrenula* species in IKI is negative (such as *Pyrenulathailandica* and *P.bahiana*) or IKI+ red/orangish (such as *P.concastroma*) or IKI+ blue (such as *P.massariospora*). This new species differs from *P.thailandica* by its IKI+ red hamathecium and an unidentified lichen substance (Aptroot 2012; Aptroot et al. 2012, 2013; Ingle et al. 2018). This new species differs from *P.bahiana* by its IKI+ red hamathecium, an unidentified lichen substance and larger ascospores, the latter 26–33(–35) × 10–13(–15) μm (Malme 1929; Aptroot 2012; Aptroot et al. 2013; Ingle et al. 2018). *P.concastroma* differs from the new species by the mostly aggregated ascomata with fused walls, but separate ostioles (Aptroot 2012; Schumm and Aptroot 2021).

#### ﻿3. Pyrenula apiculata

Taxon classificationFungiPyrenulalesPyrenulaceae

M.Z. Dou & Z.F. Jia sp. nov.

A9FAD0C7-282C-5DC0-A86A-8D1E217DE2FF

Fungal Names: FN 571678

Fig. 4

##### Diagnosis.

The new species can be distinguished from the most similar species *Pyrenulabahiana* by the absence of endospore layers in the spore tips and the absence of pseudocyphellae.

##### Type.

China. Yunnan Province: Mengla County, Xishuangbanna Tropic Botanical Garden, Chinese Academy of Sciences, Green Stone Forest, Buttress Roots, 21°54′39′′N, 101°17′05′′E, alt. 672 m, on bark, 26 January 2018, X. Zhao YN18172 (LCUF: holotype: YN18172; GenBank OR578592 for ITS and OR578573 for LSU).

##### Description.

***Thallus*** corticolous, crustose, olive-green, corticate without pseudocyphellae, UV-. ***Ascomata*** perithecioid, emergent, dispersed, conical, flattened, 0.3–0.5 mm diam., with crystals, the sides partly covered by the thallus, KOH-. ***Ostioles*** apical, black. ***Hamathecium*** not inspersed, IKI-. ***Ascospores*** 8 per ascus, uniseriate, with gelatinous halo before becoming old, 3-septate, 18–34 × 10–15 μm; middle lumina triangular to round, end lumina triangular, without layer of endospore in the spore tips; hyaline when young, reddish-brown when mature, over-mature ascospores with red oil.

**Figure 4.**
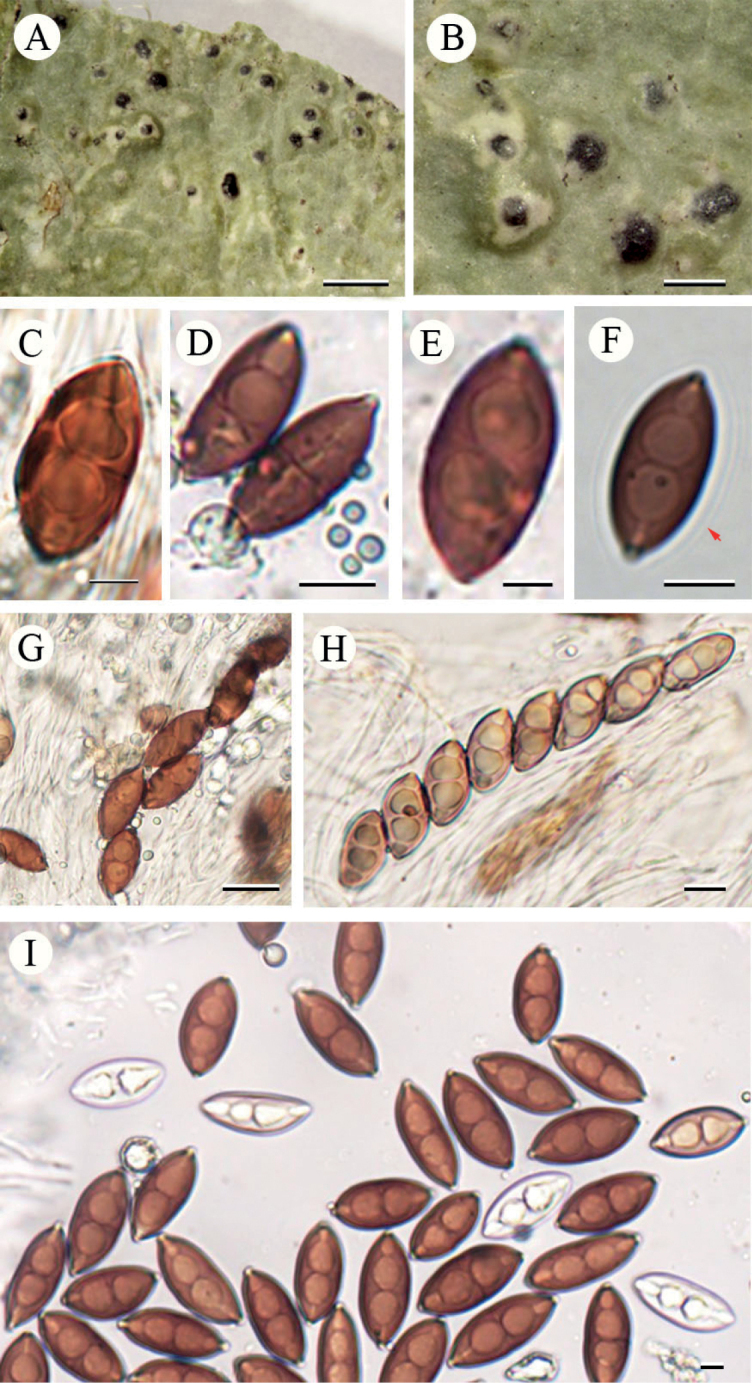
*Pyrenulaapiculata* (LCUF YN18172) **A, B** thallus with apothecia **C–E** over-mature ascospores with red oil **F–I** ascospores at different developmental stages, red arrow in **F** shows gelatinous sheath. Scale bars: 2 mm (**A**); 1 mm (**B**); 5 μm (**C, E, I**); 10 μm (**D, F, H**); 20 μm (**G**).

##### Chemistry.

Thallus K-, C-, KC-, UV-, hamathecium IKI-.

##### Ecology and distribution.

The new species is currently only known from the tropical region of southern China on bark.

##### Etymology.

The specific epithet *apiculata* refers to the pointed bulge of the end locules of ascospores.

##### Additional specimens examined.

China. Yunnan Province: Mengla County, Xishuangbanna Tropic Botanical Garden, Chinese Academy of Sciences, Green Stone Forest, Buttress Roots, 21°54′39′′N, 101°17′05′′E, alt. 672 m, on bark, 26 January 2018, X. Zhao YN18173 (LCUF, GenBank for ITS and for LSU), same locality, YN18174; China.

##### Notes.

This new species is similar to *Pyrenulathailandica*, *P.bahiana* and *P.concastroma* in having 3-septate ascospores with red or orange oil when over-mature. It differs from *P.thailandica* by the absence of pseudocyphellae, the absence of endospore layers in the spore tips and reddish-brown and smaller ascospores, which measure in the latter (30–)35–51 × (10–)14–20 μm (Aptroot 2012; Aptroot et al. 2012, 2013; Ingle et al. 2018). This new species differs from *P.bahiana* by the reddish-brown ascospores when mature, absence of endospore layers in the spore tips and absence of pseudocyphellae (Aptroot 2012; Aptroot et al. 2013; Ingle et al. 2018). *P.concastroma* differs from the new species by the mostly aggregated ascomata with fused walls, but separate ostioles (Aptroot 2012; Schumm and Aptroot 2021).

### ﻿Key to *Pyrenula* with red or orange oil in over-mature ascospores

**Table:** 

1	Ascospores transversely septate	**2**
–	Ascospores submuriform to muriform	**8**
2	Ascospores 5-septate, 22–34 × 8–14 µm	***Pyrenulasexlocularis* (Nyl.) Müll. Arg.**
–	Ascospores 3-septate	**3**
3	Ascomata mostly aggregated, with fused walls, but with separate ostioles, ascospores 31–40 × 15–16 µm	***Pyrenulaconcastroma* R.C. Harris**
–	Ascomata mostly simple, only aggregated by chance when crowded	**4**
4	Hamathecium inspersed, ascospores 28.5–50 × 10–20 μm, ascomata ca. 1–1.5 mm diam	***Pyrenulainspersa* M.Z. Dou & Z.F. Jia**
–	Hamathecium not inspersed	**5**
5	Ascospores < 35 μm long	**6**
–	Ascospores > 35 μm long	**7**
6	Terminal locules directly against the exospore wall; ascospores 18–34 × 10–15 μm; ascomata ca. 0.3–0.5 mm diam	***Pyrenulaapiculata* M.Z. Dou & Z.F. Jia**
–	Terminal locules separated from the exospore wall by endospore thickening; ascospores 26–33(–35) × 10–13(–15) µm; ascomata ca. 0.4–0.6 mm diam	***Pyrenulabahiana* Malme**
7	Hamathecium IKI-; no substances detected by TLC; ascospores (30–)35–51 × (10–)14–20 µm; ascomata ca. 0.6–1.1 mm diam	***Pyrenulathailandica* Aptroot**
–	Hamathecium IKI+ red; TLC showed an unidentified substance at Rf four of solvent C; ascospores (30–)35–55 × (12–)15–23 µm; ascomata ca. 0.8–1.6 mm diam	***Pyrenulathailandicoides* M.Z. Dou & Z.F. Jia**
8	Ascospores submuriform, the sections usually simple, the rest bicellular, 22–40 × 10–17 µm	***Pyrenulaseminuda* (Müll. Arg.) Sipman & Aptroot**
–	Ascospores muriform	**9**
9	Ascospores 25–35 × 12–13 µm, with 8 rows of 3–4 lumina per row	***Pyrenulabreutelii* (Müll. Arg.) Aptroot**
–	Ascospores 35–45 × 14–16 μm, with 8 rows of 1–3 lumina per row	***Pyrenulamacularis* (Zahlbr.) R.C. Harris**

## Supplementary Material

XML Treatment for Pyrenula inspersa

XML Treatment for Pyrenula thailandicoides

XML Treatment for Pyrenula apiculata

## ﻿Additional information

### Author ORCIDs

Jiechen Li https://orcid.org/0009-0001-8819-8407

André Aptroot https://orcid.org/0000-0001-7949-2594

### Data availability

All of the data that support the findings of this study are available in the main text or Supplementary Information.

## Supplementary materials

Supplementary material 1 ML tree showing the internal phylogeny of the family Pyrenulaceae, based on a two-gene dataset (ITS and nuLSU) and 121 taxa Data type pdf Explanation note *Cyphellophoraeuropaea* and *Endocarponpusillum* are the out-group taxa. Only significant values (higher than 70% BS) are shown. https://binary.pensoft.net/file/983181This dataset is made available under the Ope.0n.0 Database License (http://opendatacommons.org/licenses/odbl/1.0/). The Open Database License (ODbL) is a license agreement intended to allow users to freely share, modify, and use this Dataset while maintaining this same freedom for others, provided that the original source and author(s) are credited.Mingzhu Dou, Shengnan Liu, Jiechen Li, André Aptroot, Zefeng Jia

Supplementary material 2 Section of the ascomata of *Pyrenulainspersa* (LCUF HN17058) showing hamathecium with inspersion Data type jpg https://binary.pensoft.net/file/983182This dataset is made available under the Open Database License (http://opendatacommons.org/licenses/odbl/1.0/). The Open Database License (ODbL) is a license agreement intended to allow users to freely share, modify, and use this Dataset while maintaining this same freedom for others, provided that the original source and author(s) are credited.Mingzhu Dou, Shengnan Liu, Jiechen Li, André Aptroot, Zefeng Jia

Supplementary material 3 Section of the ascomata of *Pyrenulathailandicoides* (LCUF YN18212) showing hamathecium without inspersion Data type jpg https://binary.pensoft.net/file/983183This dataset is made available under the Open Database License (http://opendatacommons.org/licenses/odbl/1.0/). The Open Database License (ODbL) is a license agreement intended to allow users to freely share, modify, and use this Dataset while maintaining this same freedom for others, provided that the original source and author(s) are credited.Mingzhu Dou, Shengnan Liu, Jiechen Li, André Aptroot, Zefeng Jia

Supplementary material 4 The colour reaction of hamathecium of *Pyrenulathailandicoides* (LCUF YN18212) just in I Data type tif https://binary.pensoft.net/file/983184This dataset is made available under the Open Database License (http://opendatacommons.org/licenses/odbl/1.0/). The Open Database License (ODbL) is a license agreement intended to allow users to freely share, modify, and use this Dataset while maintaining this same freedom for others, provided that the original source and author(s) are credited.Mingzhu Dou, Shengnan Liu, Jiechen Li, André Aptroot, Zefeng Jia

Supplementary material 5 TLC test of the new species *Pyrenulathailandicoides* using C solvent systems Data type pdf https://binary.pensoft.net/file/983185This dataset is made available under the Open Database License (http://opendatacommons.org/licenses/odbl/1.0/). The Open Database License (ODbL) is a license agreement intended to allow users to freely share, modify, and use this Dataset while maintaining this same freedom for others, provided that the original source and author(s) are credited.Mingzhu Dou, Shengnan Liu, Jiechen Li, André Aptroot, Zefeng Jia

Supplementary material 6 TLC test of the new species *Pyrenulathailandicoides* using B solvent systems Data type pdf https://binary.pensoft.net/file/983186This dataset is made available under the Open Database License (http://opendatacommons.org/licenses/odbl/1.0/). The Open Database License (ODbL) is a license agreement intended to allow users to freely share, modify, and use this Dataset while maintaining this same freedom for others, provided that the original source and author(s) are credited.Mingzhu Dou, Shengnan Liu, Jiechen Li, André Aptroot, Zefeng Jia
