# Supplementary figures and images for: ﻿Three new Pyrenula species with 3-septate ascospores with red or orange oil when over-mature (Ascomycota, Pyrenulales, Pyrenulaceae) from China
Source: MycoKeys. 2024 Feb 12;102:107–25. doi: 10.3897/mycokeys.102.113619 (PMC10877525; doi:10.3897/mycokeys.102.113619)

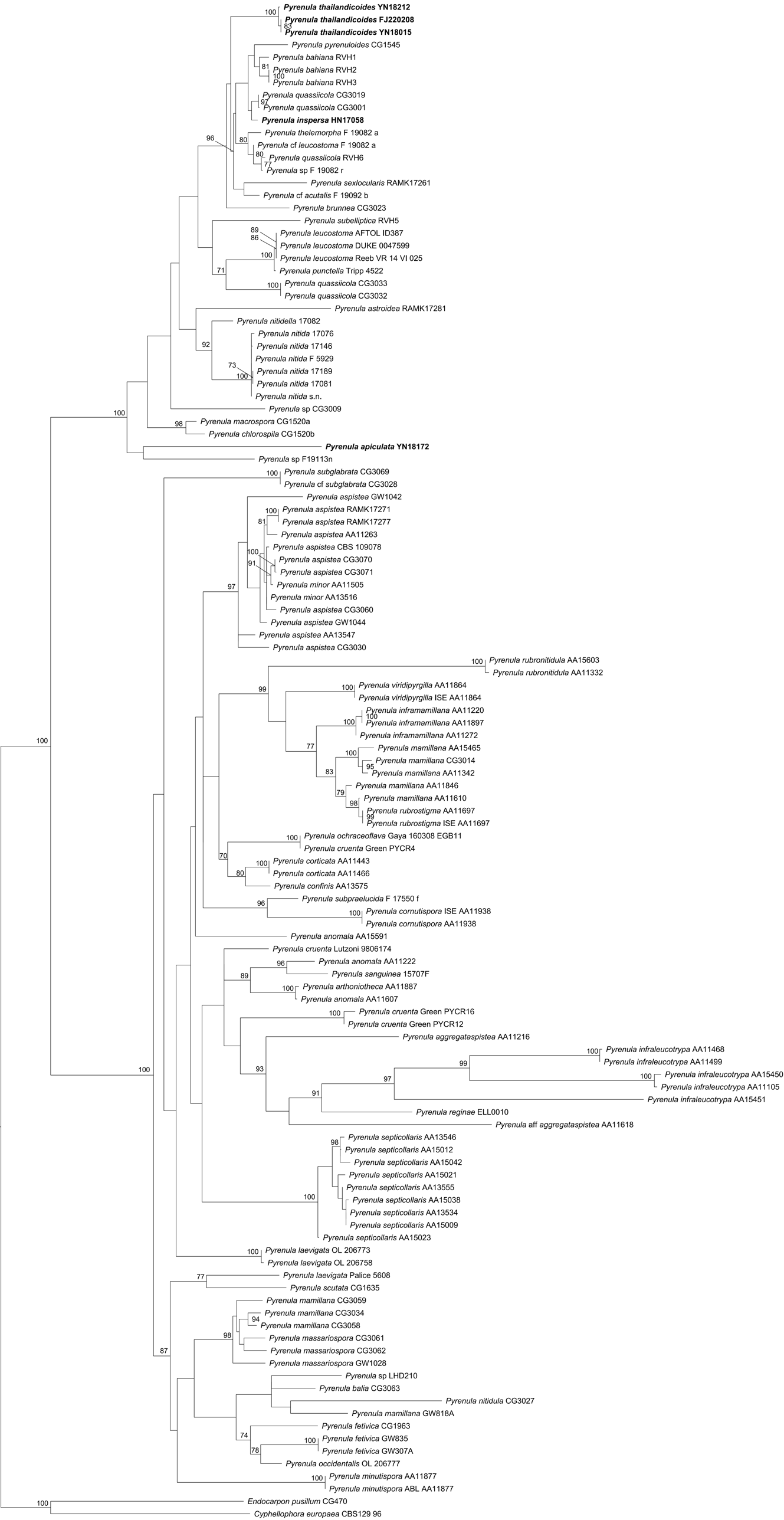

Supplement: Supplementary material 1 — ML tree showing the internal phylogeny of the family Pyrenulaceae, based on a two-gene dataset (ITS and nuLSU) and 121 taxa [file mycokeys-102-107-s001.pdf]

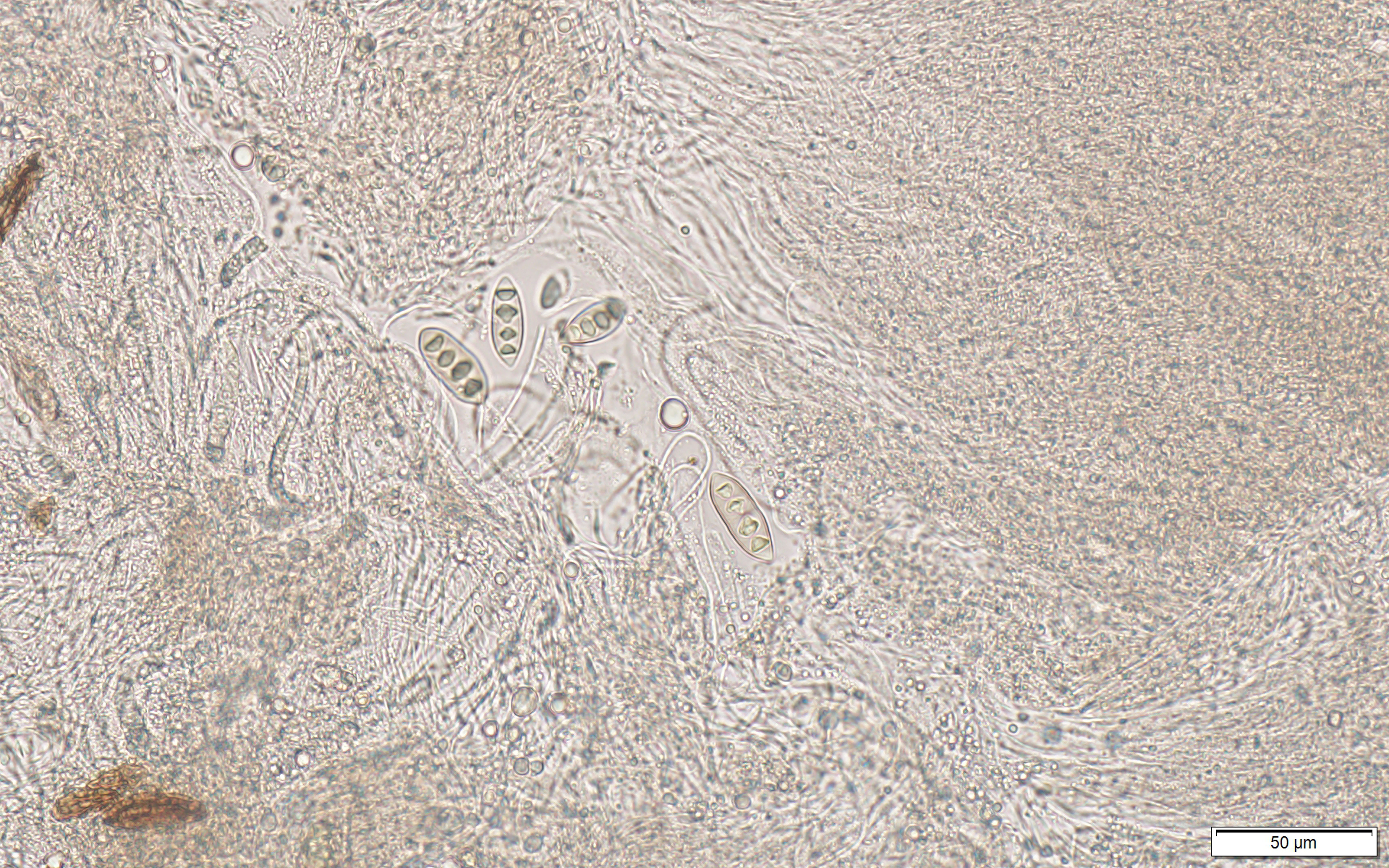

Supplement: Supplementary material 2 — Section of the ascomata of Pyrenulainspersa (LCUF HN17058) showing hamathecium with inspersion [file mycokeys-102-107-s002.jpg]

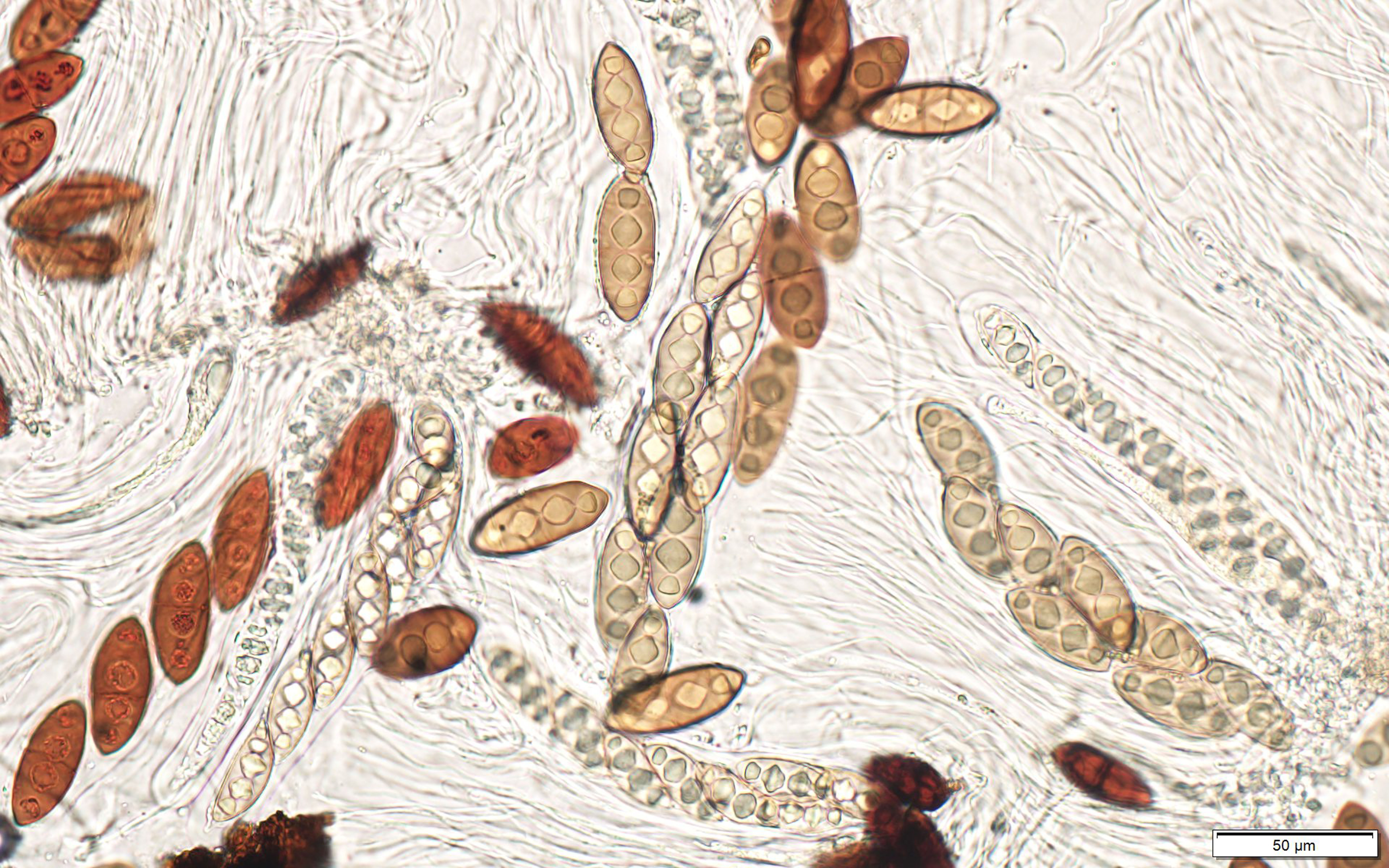

Supplement: Supplementary material 3 — Section of the ascomata of Pyrenulathailandicoides (LCUF YN18212) showing hamathecium without inspersion [file mycokeys-102-107-s003.jpg]

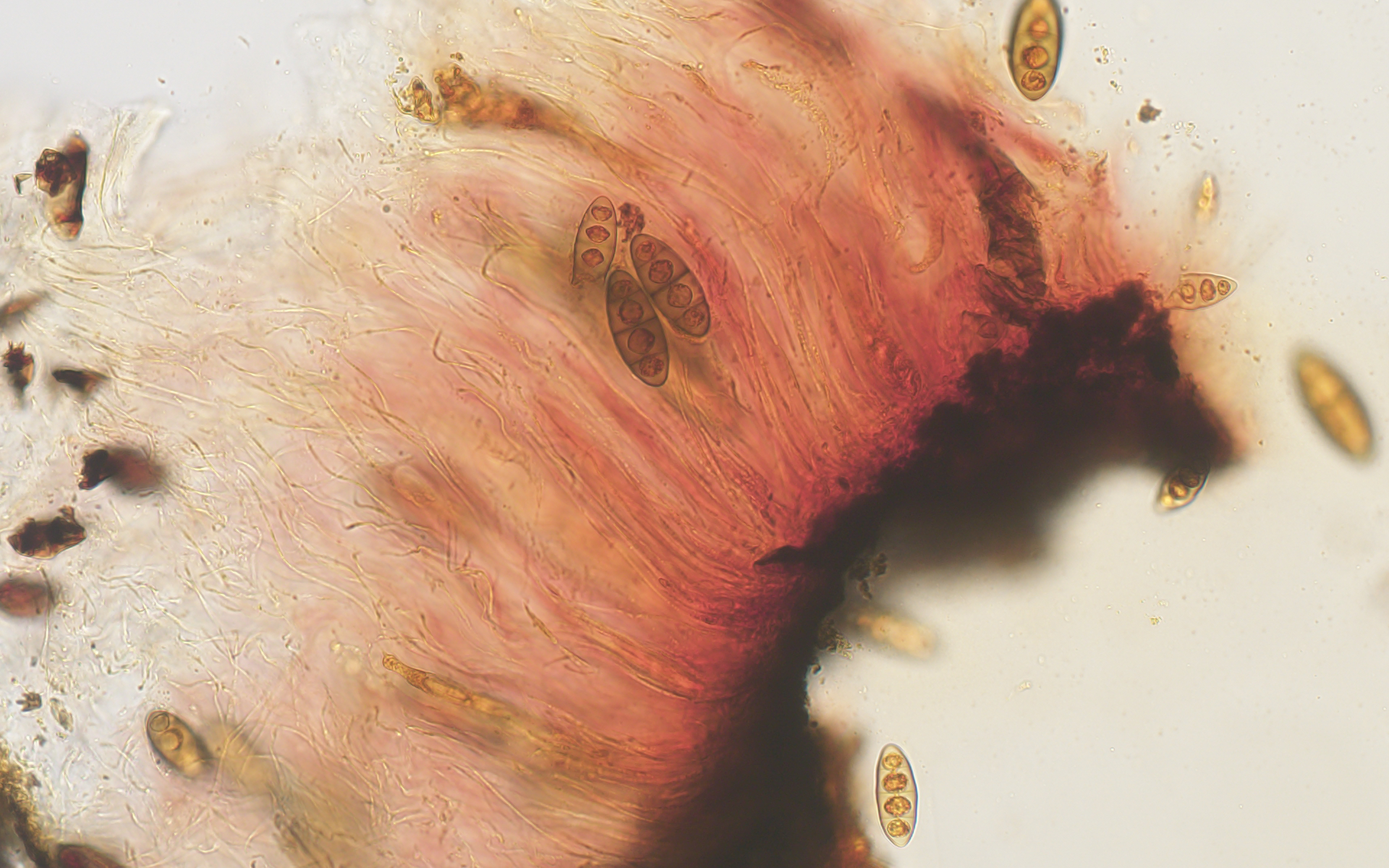

Supplement: Supplementary material 4 — The colour reaction of hamathecium of Pyrenulathailandicoides (LCUF YN18212) just in I [file mycokeys-102-107-s004.tif]
