# Supplementary material for: ﻿Three new Pyrenula species with 3-septate ascospores with red or orange oil when over-mature (Ascomycota, Pyrenulales, Pyrenulaceae) from China
Source: MycoKeys. 2024 Feb 12;102:107–25. doi: 10.3897/mycokeys.102.113619 (PMC10877525; doi:10.3897/mycokeys.102.113619)
Supplement: Supplementary material 6 — TLC test of the new species Pyrenulathailandicoides using B solvent systems [file mycokeys-102-107-s006.pdf]

Supplementary 6. TLC test of the new species *Pyrenula thailandicoides* using B solvent system. 0: standard substance (*Lethariella cladonioides*, norstictic acid at Rf four and atranorin at Rf seven). 1: The bark without thallus of the new species *Pyrenula thailandicoides* (YN18015) as the control; 2: The thallus of YN18015; 3: The bark without thallus of the new species *Pyrenula thailandicoides* (FJ220208) as the control; 4: The thallus of FJ220208; 5: The bark without thallus of the new species *Pyrenula thailandicoides* (YN18212) as the control; 6: The thallus of YN18212.

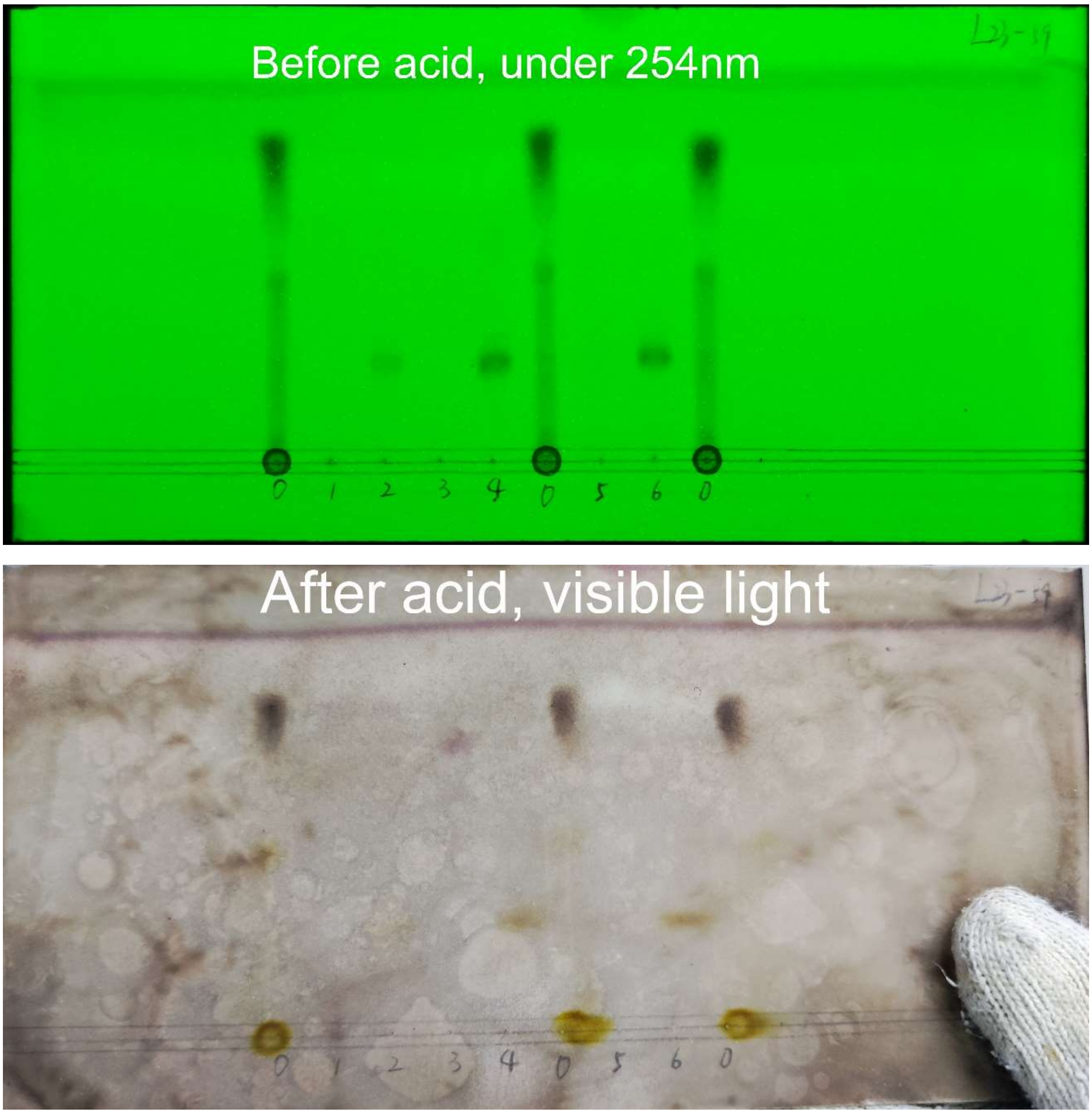

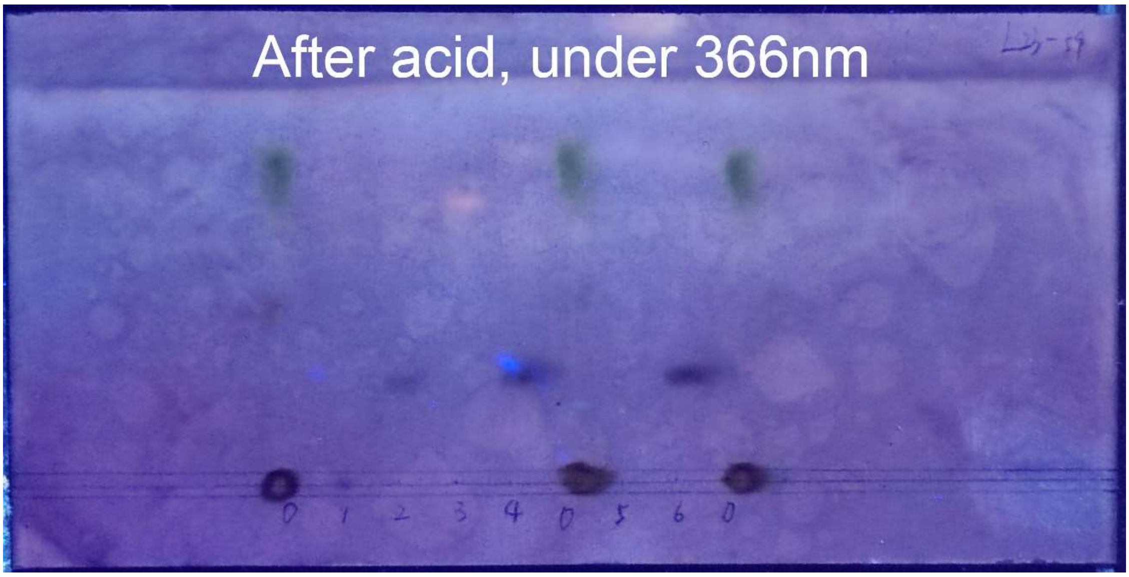
After acid, under 366nm
